# Sanitary Reform

**Published:** 1921-06-18

**Authors:** 


### THE PUBLIC WELL-BEING.

### Sanitary Reform.

At a meeting of the Institution of Sanitary Engineers an interesting discussion followed the reading by Major T. J. Moss-Flower, of Bristol, of a paper in which he dealt at some length with the many hindrances that impede the way to sanitary reform, a condition which he contended arises from ignorance, apathy, indifference, thoughtlessness, and selfishness. The Major included all parties in the State in his indictment. Were it not so, he said, we should not find hundreds of thousands of houses in the insanitary condition in which they are to-day, nor should we find so many districts in this country either entirely without reliable sources of water supply, or at the best having inferior supplies only, nor would there be thickly populated centres entirely without systems of sewerage and sewage disposal works. Yet, he submits, the absence of these requirements of modern civilisation produces an appalling condition of things "in many cases even within a short distance of the greatest city in the world." Ignorance and apathy, he points out, can be got over by education, but neglect to carry out the requirements of the Legislature could and should be dealt with according to the statute law. We quite agree that if the Major's wide but vague accusation is true it is amazing that the statute law should remain a dead letter. But what are the public sanitary engineers doing whose duty it is in behalf of the public to see that the law is carried out? Nobody is going to say that the British sanitary system is perfect, or that it is not applied very much less efficiently in some places than in others. But does Major Moss-Flower seriously maintain that there are in England to-day "thickly populated centres entirely without systems of sewerage and sewage disposal works?" If so, where are they, and why does he not name them for the good of the community and the information of a Ministry of Health which would be only too glad to have the facts at their disposal? We must confess to some doubt of Major Moss-Flower's critical qualifications when we find that he appears to attribute the "appalling" state of things sanitary to the national love of sport and to the growing luxury in dress! It must be rather difficult for the plain man to detect the relationship between cricket, beautiful clothes, and sanitary reform, unless the argument be that if John Smith did not play cricket or that Mary Jones was not concerned about jumpers they would both pay immediate attention to the question of sanitary reform. It requires some stretch of the imagination to believe that to be possible, but the curious thing is that the Major from Bristol so far hypnotised his audience of fellow sanitary experts that instead of discussing sanitation they proceeded to debate the place of sport in national life and the "crime" that multitudes of women commit (according to the phrase used by yet another sanitary major) in "flaunting expensive jewellery in the faces of starving unemployed." It was the oddest as well as the most amusing debate we have ever read by a body of experts who came together ostensibly for the purpose of elucidating the problems of their profession. But we are afraid it will not do much to assist the cause of sanitary reform.

